# Evaluation of genomic characteristics of *Escherichia coli* associated with bovine mastitis and the potential utility of alternative antimicrobial methods

**DOI:** 10.1007/s42770-026-02040-5

**Published:** 2026-07-29

**Authors:** Jônathas Moreno Silva de Souza, Gabryel Bernardo Vieira de Lima, Daniela dos Santos Cassimiro, Sheila da Silva, Thiago de Jesus Sousa, Danillo Sales Rosa, Renata de Faria Silva Souza, Fernando Antônio Gomes da Silva Júnior, Helinando Pequeno de Oliveira, Sílvia Cristina Osaki, Vasco Ariston de Carvalho Azevedo, Bertram Brenig, Flavia Figueira Aburjaile, Mateus Matiuzzi da Costa

**Affiliations:** 1 Universidade Federal de Minas Gerais (UFMG), Belo Horizonte, 31270-010 Brazil; 2 Universidade Federal do Vale do São Francisco (UNIVASF), Petrolina, Pernambuco 56300-000 Brazil; 3 Universidade Federal do Rio de Janeiro (UFRJ), Rio de Janeiro, 22290-90 Brazil; 4 Laboratório Central de Saúde Pública do Espírito Santo, Vitória, 29017-010 Brazil; 5 Universidade Federal Rural de Pernambuco (UFRPE), Recife, 52171-900 Brazil; 6 Universidade Federal do Paraná (UFPR), Palotina, 85950-000 Brazil; 7 University of Göttingen, Göttingen, 37073 Germany

**Keywords:** Bactericidal, Bioinformatics, Cattle farming, *E. coli*, Resistance genes

## Abstract

Mastitis is a disease that affects dairy cattle and can be caused by multidrug-resistant *Escherichia coli*, causing losses to the agricultural sector. To address this, research into new substances with antimicrobial activity is crucial. Thus, the objective of this study was to evaluate the efficacy of extracts of *Commiphora leptophloeos*, *Amburana cearensis*, *Agave sisalana*, and highly soluble polypyrrole (Hs-PPy) against *E. coli* isolates. Eight *E. coli* isolates from bovine mastitis and a standard strain were used. The presence of efflux pumps was investigated by ethidium bromide extrusion. Sequencing was performed on the Illumina HiSeq 2500 platform using a 2 × 150 bp library with quality assessed via FastQC and assembly completed using Unicyler. Genome annotation and the identification of resistance and virulence genes were carried out using PROKKA and PanViTa, respectively. Species confirmation as *E. coli* was achieved via Jspecies and pyANI public genomes from NCBI. Finally, antimicrobial activity was determined by broth microdilution. Among the natural products tested against the isolates only *A. cearensis* showed antibacterial activity, with a concentration of 12,500 µg/mL, whereas Hs-PPy showed activity at 250 µg/mL. The isolates tested positive for the presence of efflux pump genes, but only 3 isolates expressed the mechanism in vitro. The natural products were not effective in eliminating the bacteria. Hs-PPy was the only compound that demonstrated bactericidal activity against the bacteria, proving to be an alternative for bactericidal action, even in the presence of genes that inhibit its action against a wide variety of compounds.

## Highlights

Hs-PPy inhibited the growth of mastitis E. coli isolates at MIC 250 μg/mL;

Presence of resistance and virulence genes in mastitis-causing bacteria;

Presence of an efflux pump

## Introduction

Bovine mastitis is a common disease characterized by inflammation of the mammary gland, resulting in altered milk quality, physical pain, and substantial economic losses for dairy farms. *Escherichia coli* is a Gram-negative bacterium commonly found in the environment and the microbiota of animals and humans. It is a well-known opportunistic pathogen and a major cause of both clinical and subclinical mastitis [1–3]. Furthermore, because dairy cattle are also utilized for meat production, the presence of these foodborne pathogens poses a significant public health risk through potential consumption of contaminated beef products [4].

*E. coli* frequently adapts to invade, colonize, and proliferate within the host’s mammary gland, thereby establishing infection. Due to the resistance factor that overlaps the action of the immune system, severe infections can lead to cattle mortality. Multidrug-resistance is the major driver of lethal outcomes, a crisis heavily accelerated by the inappropriate use of antibiotics in both human and veterinary medicine. For instance, in the United States, approximately 70% of antibiotics are used within these sectors [5]. Among the virulence factors, the following stand out: first, adhesion, which promotes attachment to the mammary gland wall as the initial step in the pathogen’s colonization, thus ensuring its persistence in the bovine host. Second, motility is associated with the ability to swim and agglutinate, which promotes the proliferation of bacteria throughout the host, thereby accessing entry points into the bovine body and enabling movement within the organism. This plays a significant role in the mammary gland, as it is the most affected during bacterial infection, in addition to the normal motility of the bacteria that ensures movement toward the gradient of higher chemical concentration. Finally, the acquisition of iron from siderophores, which are reaction metabolites, constitutes one of the causes of bacterial presence in cells and tissues; iron is essential as a compound utilized by bacteria, and its concentration in the host body is not high [6–9].

The presence of efflux pumps exacerbates antibiotic resistance. These pumps are divided into six groups based on their mode of action. These pumps help transport drug compounds out of the bacterial cell, preventing antimicrobials from reaching the intracellular concentration required to eliminate the pathogen and thereby promoting bacterial survival [10]. The presence of efflux pumps in bovine mastitis isolates is therefore concerning, as it suggests the need for alternatives to the antibiotics frequently used to combat disease.

Alternative measures are important to eliminate multidrug-resistant bacteria; among these options, plants used in traditional medicine have gained attention due to their antimicrobial properties [11]. In the Brazilian Caatinga biome, *Amburana cearensis* (Allemão) A.C.Sm. has been reported to exert antibacterial effects and inhibit bacterial growth [12]. It also act as a resistance modifier, potentially displaying synergistic effects when applied alongside antibiotics or other antimicrobial substances against resistant strains [13, 14]. Furthermore, this species has demonstrated antiproliferative, anti-inflammatory, and antibacterial activities [15]. The species *Commiphora leptophloeos* (Mart.) J.B. Gillett is native to Brazil and can be found in the Caatinga, Cerrado, and Amazon biomes [16]. This plant contains active compounds, including flavonoids, phenolic acids, and tannins, which are related to its antibacterial activity against multidrug-resistant isolates [17], as well as inhibition of biofilm formation [18] and anti-inflammatory action [19].

Meanwhile, the species *Agave sisalana* Perrine ex Engelm. is found in tropical climates, with better development in semi-arid regions, and contains saponins, which are glycosides from the plant’s secondary metabolism with antibacterial action against Gram-negative bacteria such as *E. coli* [20, 21]. In addition, the plant extract also exhibits an antibiofilm action against the same group [22]. Previous studies suggest that highly soluble polypyrrole (Hs-PPy) is a positively charged conducting polymer that acts as a bactericidal agent by breaking the lipid membrane of bacteria, and its use in nanoparticles has shown antibacterial activity against Gram-positive and Gram-negative bacteria [23–25]. Therefore, this study aims to evaluate the efficacy of three plant extracts and Hs-PPy against eight *E. coli* isolates from bovine mastitis, which were subjected to phenotypic and molecular characterization of virulence factors and antimicrobial resistance.

## Materials and methods

### Bacterial isolates

Eight *E. coli* isolates from bovine mastitis were used. These isolates were obtained from bovine milk samples between February 2020 and June 2020 at the experimental farm (24.174440 N 53.503143 W) of the Federal University of Paraná, Palotina, Brazil. The cultivation was carried out using milk samples according to the National Mastitis Council [26]. The *E. coli* strain from the American Type Culture Collection, ATCC 25922, was selected as a control for antimicrobial susceptibility and the presence of efflux pumps. The isolates were registered on the platform of the National System for the Management of Genetic Heritage and Associated Traditional Knowledge (SISGEN) under the code AD52443. This study was approved by the Ethics Committee on Animal Use of the Federal University of Paraná (UFPR) under the number 50/2014.

### Antimicrobial susceptibility test

Nine antimicrobials from different classes were tested against *E. coli*: amoxicillin (10 mcg), ampicillin (2 mcg), azithromycin (15 mcg), cephalexin (30 mcg), enrofloxacin (5 mcg), neomycin (30 mcg), sulfazotrim (25 mcg), and tetracycline (30 mcg). Tests were performed in compliance with the standards determined by the Clinical and Laboratory Standards Institute [27].

For this purpose, an aliquot of the bacterial suspension (~ 1.5 × 10^8^ CFU/mL) was swabbed onto Mueller-Hinton (MH) agar plates before the antibiotic discs were added. The plates were then incubated at 37 °C for 24 h. The multidrug antibiotic resistance (MAR) index was calculated according to the equation below [28].

$$MAR\;=\;\frac{N.\;\underline\circ\;of\;resistant\;antibiotics}{N.\;\underline\circ\;of\;antibiotics\;tested\;}$$

### Phenotypic detection of efflux pumps

The isolates were previously grown on Brain Heart Infusion (BHI) agar plates. *Staphylococcus aureus* 1199 was used as a negative control and *S. aureus* 1199B as a positive control. For isolate 1199B, the culture medium was supplemented with 16 µg/ml of norfloxacin.

Subsequently the MH plates were prepared with 0.5 µg/mL of ethidium bromide, and the plate was spiked with a disposable inoculation loop [29]. The plate was then incubated in a bacteriological oven at 37 °C for 24 h and read using a transilluminator.

### Sequencing, assembly, quality and annotation

Previously extracted DNA was sequenced on the Illumina HiSeq 2500 platform using a 2 × 150 bp library. Quality control was performed with FastQC v0.12.1 [30]. The assembly was generated with Unicycler v0.4.8 [31], with a parameter that limits the contigs to a minimum size of 200 nucleotides. The quality of the assemblies was checked with Quast v5.0.2 [32]. Subsequent analyses were also carried out to check the bias using CheckM2 v1.1.0 [33]., while BUSCO v5.4.7 (Benchmarking Universal Single-Copy Orthologs) [34] was applied to estimate genome completeness analyses against the Enterobacterales Order. Finally, functional annotation was performed using PROKKA v1.14.6 (Prokaryotic Genome Annotation) [35]. All the genomes of the isolates were deposited under Bioproject PRJNA1182684 at NCBI (National Center for Biotechnology Information).

### Taxonomy

The genomes used were isolated genomes and public genomes from NCBI, which were filtered for integrity to only include isolates with complete, referenced, and annotated genomes from the period between 2010 and 2025, totaling 3.736 genomes. Subsequently, genomes for which information on the collection site, sample type, and host—not limited to cows—was unavailable were excluded, resulting in a final total of 199 genomes.

Two different analyses were used for taxonomic confirmation. The Tetra Correlation Search (TCS) was used only with the isolates from the study and performed on the online platform JSpecies (available at <https://jspecies.ribohost.com/jspeciesws/%3E), which provides a z-score result for the species. An Average Nucleotide Identity analysis with MUMmer (ANIm) was performed using pyANI v0.2.12 [36] to assess nucleotide identity among all genomes, using 207 *E. coli*, 10 *Shigella* sp., and 10 *Klebsiella* sp. references as an external group.

### Resistome and virulome

PanViTa v1.1.5 [37, 39] was used to screen the isolates for resistance and virulence genes, using a threshold of 70% identity and coverage. Resistance profiles were evaluated against the Comprehensive Antibiotic Resistance Database (CARD) [38], while virulence factors were identified using the Virulence Factor Database (VFDB) [39].

### Obtaining and diluting plant extracts and compounds

For the leaves of *A. cearensis* (Allemão) A.C.Sm. (SisGen, registration number A9F02E1), the extract was prepared according to the method described by Gouveia et al. [40]. The concentration was determined to be 25,000 µg/mL, with the extract being weighed and diluted with distilled water, after which the solution was stored at 4 °C. Extraction of *C. leptophloeos* (Mart.) J.B.Gillett. leaves (SisGen registration number AE82EC3) followed the methodology of Wagner and Bladt [41]. This extract was diluted to 25,000 µg/mL using a solution of 10% Tween and 90% distilled water, homogenized in an ultrasonic bath and stored at 4 °C. Similarly, the ethanolic extract of *A. sisalana* Perrine ex Engelm was prepared at an initial stock concentration of 100,000 µg/mL. It was diluted using the same 10% Tween and 90% distilled water ratio, homogenized in an ultrasonic and stored at 4 °C.

Hs-PPy was obtained according to Da Silva et al. [42], with reduced pressure purification, where sodium dodecyl sulphate (SDS) is immersed in milli-Q water and then pyrrole is added. The solution was then stirred for 45 min, followed by the addition of ammonium persulphate by dripping, still stirring for 35 min, and finally stored at 4 °C for 24 h, at a concentration of 2,000 µg/mL.

### Antimicrobial activity by broth microdilution

The minimum inhibitory concentration (MIC) and minimum bactericidal concentration (MBC) were determined by broth microdilution according to protocol M07-A11 [27]. Initially, 100 µL of MH broth was added to the wells of a 96-well microplate. Then, 100 µL of the ethanolic extract of *A. cearensis*, *C. leptophloeos*, *A. sisalana* or Hs-PPy were added separately to three wells of the first row of the microplate and serial microdilution (1:2) was performed. Subsequently, 10 µL of a bacterial suspension at ~ 1.5 × 10^6^ CFU/mL was added to the wells. Sterile MH broth was used as a sterility control, and MH broth plus the bacterial suspension was used as a bacterial viability control. The microplate was incubated at 37 °C for 24 h.

The contents of the microplate were transferred to a plate containing MH agar, using a replicator, followed by incubation at 37 °C for 24 h. The MBC was determined from the visual reading of bacterial growth on the plate and considered to be the lowest concentration of the substance capable of killing the bacteria. To determine the MIC, 20 µL of 1% 2,3,5-triphenyltetrazolium chloride (TTC) (ACS) was added to each well of the microplate and incubated at 37 °C for 1 h. The change in color to a reddish hue was considered indicative of bacterial viability. The MIC was the lowest concentration capable of inhibiting bacterial growth. The tests were performed in technical and biological triplicate.

### Statistical analysis

The MIC data were analyzed using the Friedman non-parametric multiple comparison test with Dunn’s post-test. Statistical analyses were performed using GraphPad Prism 8 software, and results were plotted as mean ± standard deviation. A p-value of < 0.05 was used for all tests.

## Results

### Antimicrobial susceptibility test

**Fig. 1:**
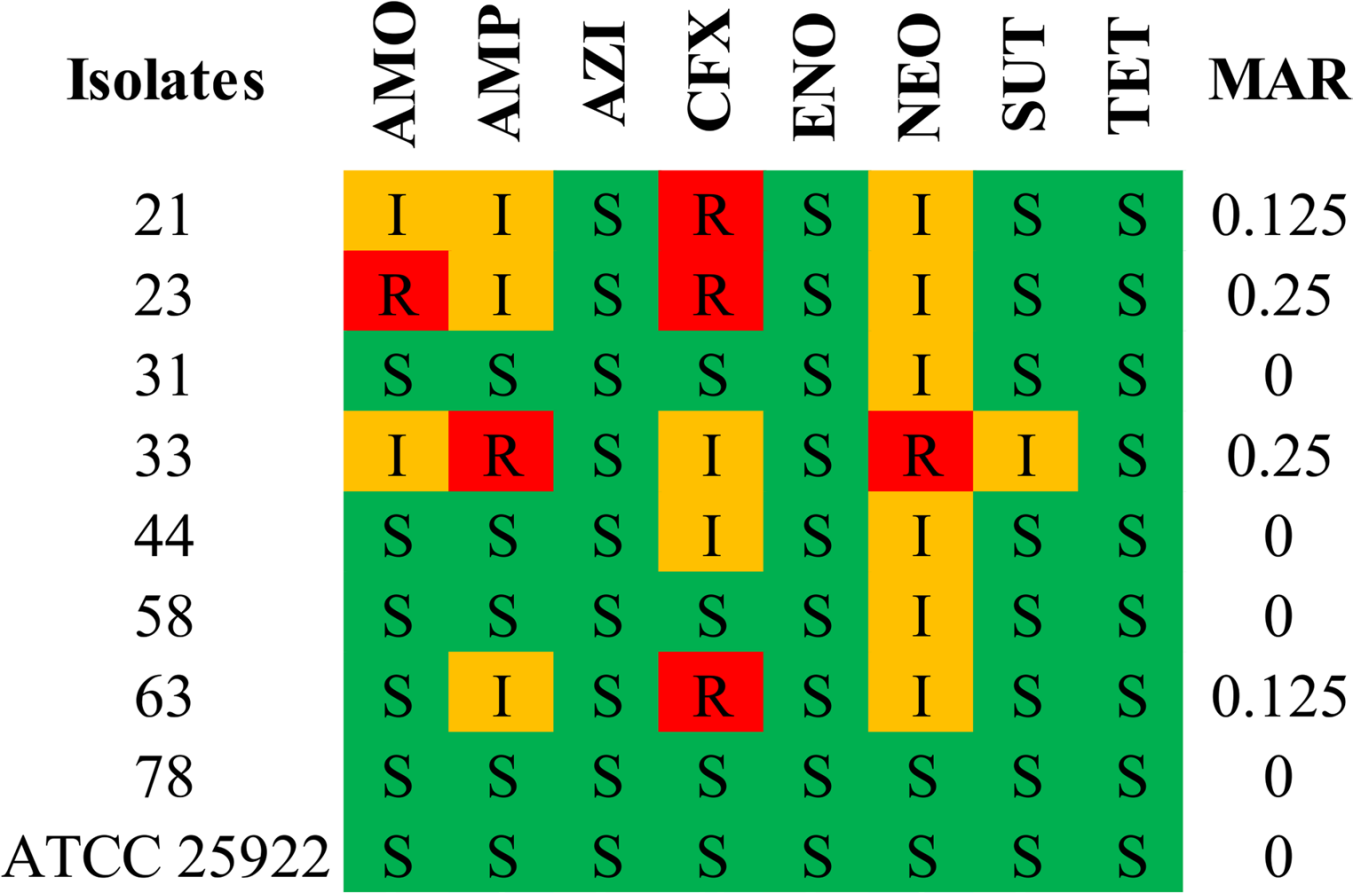
Antimicrobial susceptibility profile of *Escherichia coli* isolates from bovine mastitis. Amoxicillin (AMO), ampicillin (AMP), azithromycin (AZI), cephalexin (CFX), enrofloxacin (ENO), neomycin (NEO), sulfazotrim (SUT) and tetracycline (TET). Sensitive (S), intermediate (I) and resistant (R). Index of multiple resistance to antimicrobials (MAR). American Type Culture Collection (ATCC)

The isolates showed susceptibility to the majority of the antibiotics tested. No isolate was classified as multidrug-resistant, as they did not exhibit resistance to three or more classes of antimicrobials [43], with a MAR index ranging from 0 to 0.25 among the isolates. Isolates 23 and 33 showed an index of 0.25, and isolates 21 and 63 showed an index of 0.125 [28] (Fig. 1).

### Phenotypic detection of efflux pumps

**Fig. 2:**
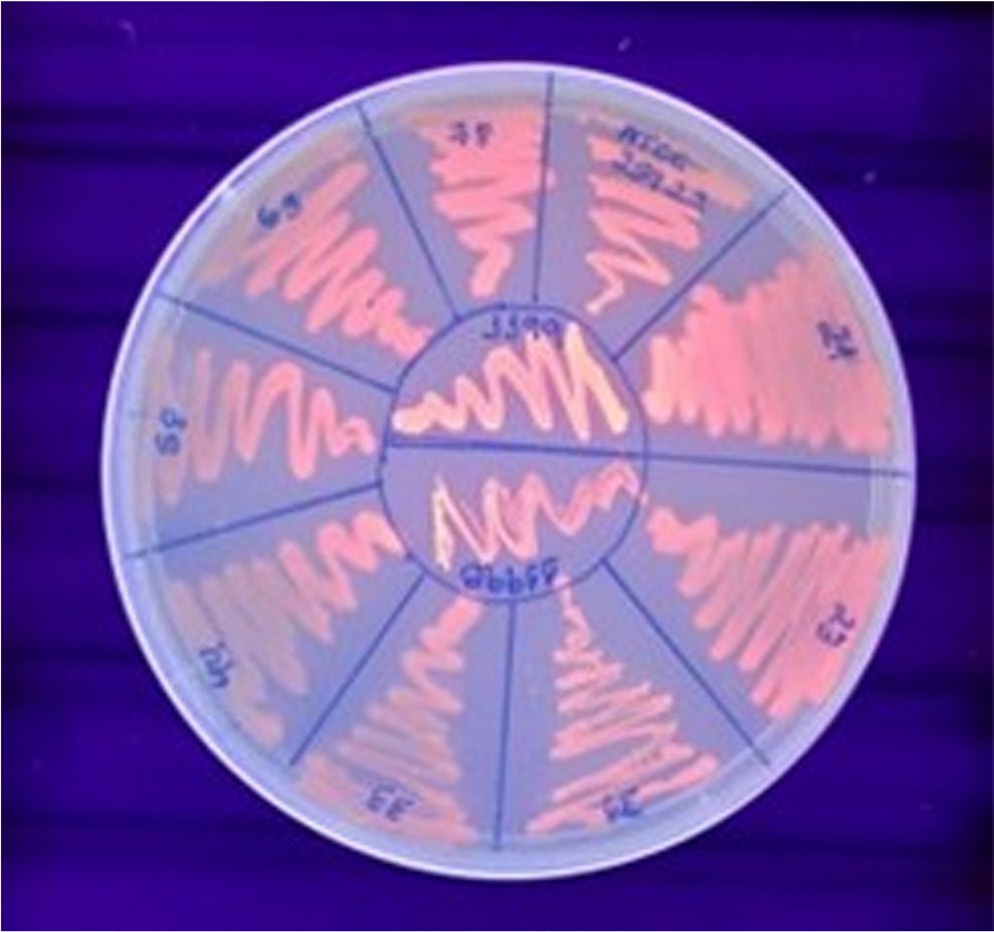
Active efflux pump activity in *Escherichia coli* isolates from bovine mastitis. The plate displays Isolate 1199 (deficient control), isolate 1199B (proficient control in efflux pump expression), ATCC 25922, and bovine mastitis isolates proficient in expression. The presence of fluorescence in the sample demonstrates the accumulation of ethidium bromide, while its absence in most of the culture streaks demonstrates the efflux of the compound from the bacterial cells

The results show that isolates 33, 44 and 58 may have efflux pumps, while the other isolates and ATCC 25922 do not have efflux pumps (Fig. 2). The absence of fluorescence in the positive isolate (1199B) can be seen in Fig. 2.

### Assembly

The assembled genomes exhibited high-quality metrics with high consistency across all assemblies, as in Table 1. Genomic quality evaluation demonstrated a maximum contamination level of 0.1%.

**Table 1:** Quality metrics for the assemblies

Isolates	21	23	31	33	44	58	63	78
%GC	50.52	50.52	50.85	50.52	50.52	50.34	50.52	50.34
N50	242,237	242,237	279,878	242,237	242,237	617,006	242,237	617,006
L50	6	6	6	6	6	4	6	4
Size (bp)	4,838,899	4,838,836	4,858,291	4,838,907	4,837,865	4,811,420	4,838,256	4,811,531
Nº Contigs	74	74	62	74	74	51	74	52

### Taxonomy

Species-level classification performed with JSpecies confirmed that all isolates belonged to *E. coli*, as expected, yielding Z-score values greater than 99.9%.

**Fig. 3:**
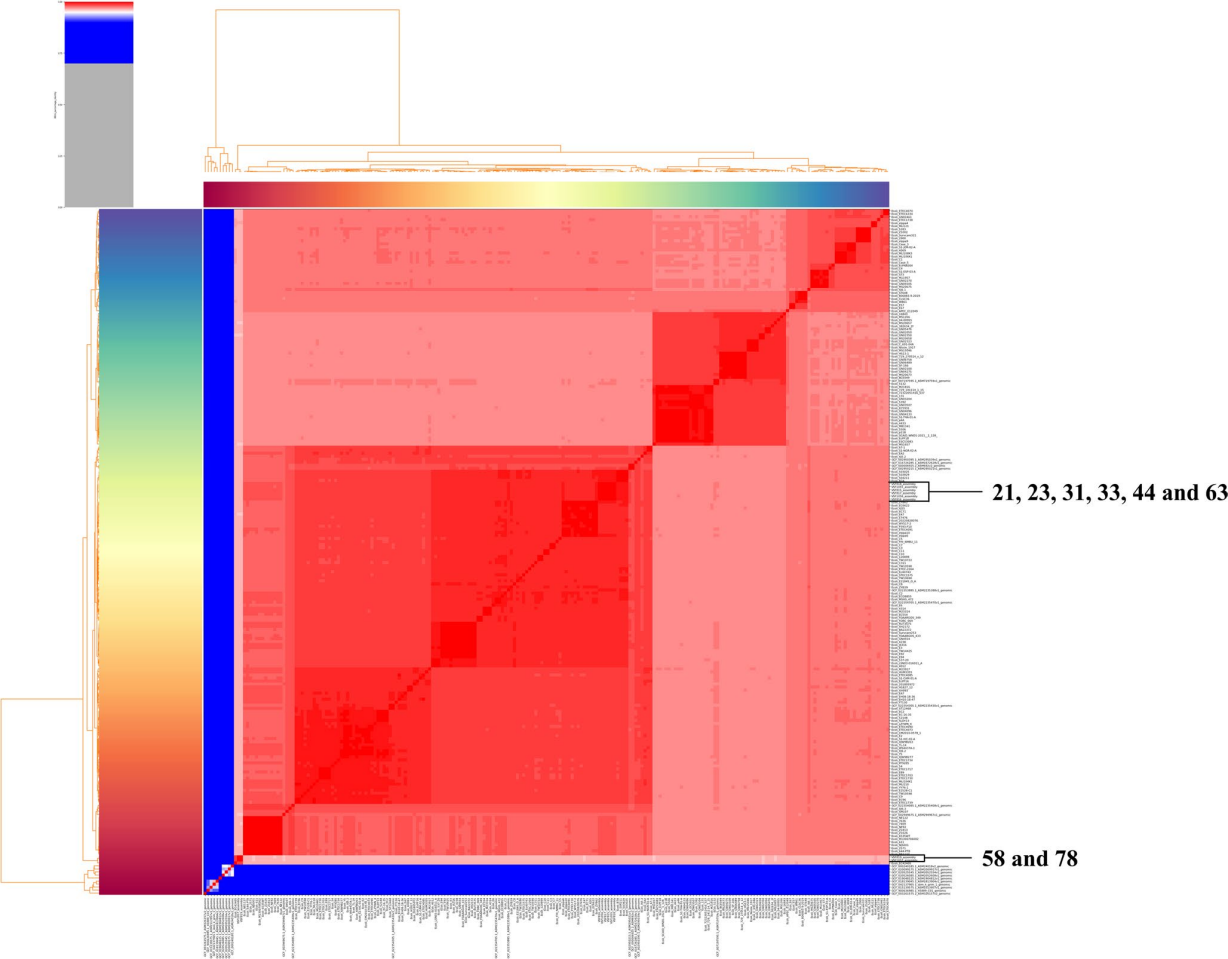
ANI heatmap of genomes isolates bovine mastitis, genomes of NCBI of *Escherichia coli* and the control *Staphylococcus chromogenes*

ANI analysis was visualized via, a heatmap (Fig. 3) comparing the identity between all study isolates and public genomes that passed the completeness filter, with all results exceeding the 95% species threshold. The clustering patterns reveal the formation of three large groups and one small group with no genomes displaying low identity relative to the others. This high genomic identity is reflected by the color gradient intensity where darker red indicates higher similarity. The isolated areas are highlighted with arrows indicating their specific locations.

### Resistome and virulome

Genes related to the efflux pump were identified across the isolates. These included *msbA*, whose function is related to ATP-linked antibiotics. Members of major facilitator superfamily (MFS) the efflux pump of antibiotics, such as emrB, emrR, emrY, evgA, H-NS, mdtG, and mdtH, as well as complete efflux pumps that are important for expelling compounds from the bacterial cell, which are listed in Table 2.

**Table 2:** Resistance factors related to antimicrobial agents identified in *Escherichia coli* isolates from bovine mastitis. Major Facilitator Superfamily (MFS), Resistance nodulation division (RND) and Small multidrug resistance (SMR)

Antimicrobial	Resistance	Family of efflux pump	Gene products
Multidrug-resistant	AcrAB-TolC	RND	AcrA represents the periplasmic portion of the transport protein.
			AcrB functions as a herterotrimer which forms the inner membrane component and is primarily responsible for substrate recognition and energy transduction by acting as a drug/proton antiporter.
			TolC is a protein subunit of many multidrug efflux complexes in Gram-negative bacteria. It is an outer membrane efflux protein and is constitutively open. Regulation of efflux activity is often at its periplasmic entrance by other components of the efflux complex.
	AcrAD-TolC	RND	AcrD is an aminoglycoside efflux pump expressed in *E. coli*. Its expression can be induced by indole, and is regulated by baeRS and cpxAR
	EmrAB-TolC	MFS	EmrA is a membrane fusion protein, providing an efflux pathway.
			EmrB is a translocase.
	KpnEF	SMR	Mutation in KpnEF resulted in increased susceptibility to cefepime, ceftriaxon, colistin, erythromycin, rifampin, tetracycline, and streptomycin as well as enhanced sensitivity toward sodium dodecyl sulfate, deoxycholate, dyes, benzalkonium chloride, chlorhexidine, and triclosan.
	MdtABC-TolC	RND	MdtA is the membrane fusion protein.
			MdtB is a transporter that forms a heteromultimer complex with MdtC to form a multidrug transporter.
			MdtC is a transporter that forms a heteromultimer complex with MdtB
	MdtNOP	MFS	MdtN is a multidrug resistance efflux pump. Could be involved in resistance to puromycin, acriflavine and tetraphenylarsonium chloride.

Genes associated with the virulome included those involved in adhesion, motility, and antimicrobial activity, which are important factors for host colonization and proliferation. These genes are listed in Table 3.

**Table 3:** Virulence genes identified in *Escherichia coli* isolates from bovine mastitis and their respective mechanisms

Virulence mechanism	Number of genes	Genes
Adherence	28	*cfaA*,* cfaB*,* cfaC*,* cfaD/cfaE*,* cgsD*,* cgsE*,* cgsF*,* cgsG*,* csgC*,* fdeC*,* fimA*,* fimB* ,*fimC*,* fimD*,* fimE*,* fimF*,* fimG*,* fimH*,* fimI*,* fimZ*,* htpB*,* lpxC*,* yagV/ecpE*, *yagW/ecpD*,* yagX/ecpC*, *yagY/ecpB*,* yagZ/ecpA* and *ykgK/ecpR*
Motility	25	*cheA*,* cheB*,* cheR*,* cheW*, *cheY*,* cheZ*,* flgB*,* flgC*,* flgD*, *flgG*,* flgH*,* flgI*,* flhA*,* flhC*,* flhD*,* fliA*,* fliG*,* fliI*,* fliM*, *fliN*,* fliP*,* fliQ*,* flmH*,* motA* and *motB*
Antimicrobial activity/ Competitive advantage	2	*acrA* and *acrB*

### Antimicrobial activity by broth microdilution

The ethanolic extract of *C. leptophloeos* did not show bacteriostatic or bactericidal activity at any of the concentrations tested against *E. coli* isolates, but it showed a MIC of 6,250 µg/mL and an MBC of 2,500 µg/mL for the standard strain. The ethanolic extract of *A. sisalana* showed MIC and MBC only at the highest concentration tested (50,000 µg/mL), both for the isolates and for the standard strain. The ethanolic extract of *A. cearensis* showed MIC and MBC of 12,500 µg/mL for all isolates, except for the standard strain, for which the MIC was 3,125 µg/mL and MBC was 6,250 µg/mL. Hs-PPy showed the best antimicrobial activity, with MIC and MBC of 250 µg/mL for all isolates and the standard strain. The concentrations were identical among the isolates and can be seen in Table 4.

**Table 4:** Antimicrobial activity of the ethanolic extracts of *Commiphora leptophloeos*, *Amburana cearensis* and *Agave sisalana*, and highly soluble polypyrrole (Hs-PPy) against *Escherichia coli* isolates from bovine mastitis

Isolates	C. leptophloeos		A. cearensis		A. sisalana		Hs-PPy	
	MIC (µg/mL)	MBC (µg/mL)	MIC (µg/mL)	MBC (µg/mL)	MIC (µg/mL)	MBC (µg/mL)	MIC (µg/mL)	MBC (µg/mL)
21	-	-	12,500 ± 0	12,500 ± 0	44,444 ± 10,393	50,000 ± 0	250 ± 0	250 ± 0
23	-	-	12,500 ± 0	12,500 ± 0	44,444 ± 10,393	50,000 ± 0	250 ± 0	250 ± 0
31	-	-	12,500 ± 0	12,500 ± 0	50,000 ± 0	50,000 ± 0	250 ± 0	250 ± 0
33	-	-	11,806 ± 1,964	12,500 ± 0	50,000 ± 0	50,000 ± 0	250 ± 0	250 ± 0
44	-	-	12,500 ± 0	12,500 ± 0	50,000 ± 0	50,000 ± 0	250 ± 0	250 ± 0
58	-	-	12,500 ± 0	12,500 ± 0	50,000 ± 0	50,000 ± 0	250 ± 0	250 ± 0
63	-	-	12,500 ± 0	12,500 ± 0	47,222 ± 7,856	50,000 ± 0	250 ± 0	250 ± 0
78	-	-	12,500 ± 0	12,500 ± 0	50,000 ± 0	50,000 ± 0	250 ± 0	250 ± 0
ATCC 25922	3,125 ± 0	6,250 ± 0	3,125 ± 0	6,250 ± 0	50,000 ± 0	50,000 ± 0	250 ± 0	250 ± 0

American Type Culture Collection (ATCC). Minimum bactericidal concentration (MBC). Minimum inhibitory concentration (MIC). (-) There was no activity even at the highest concentrations tested, which was 12,500 µg/mL for *C. leptophloeos*. The data were expressed as mean ± standard deviation

**Fig. 4:**
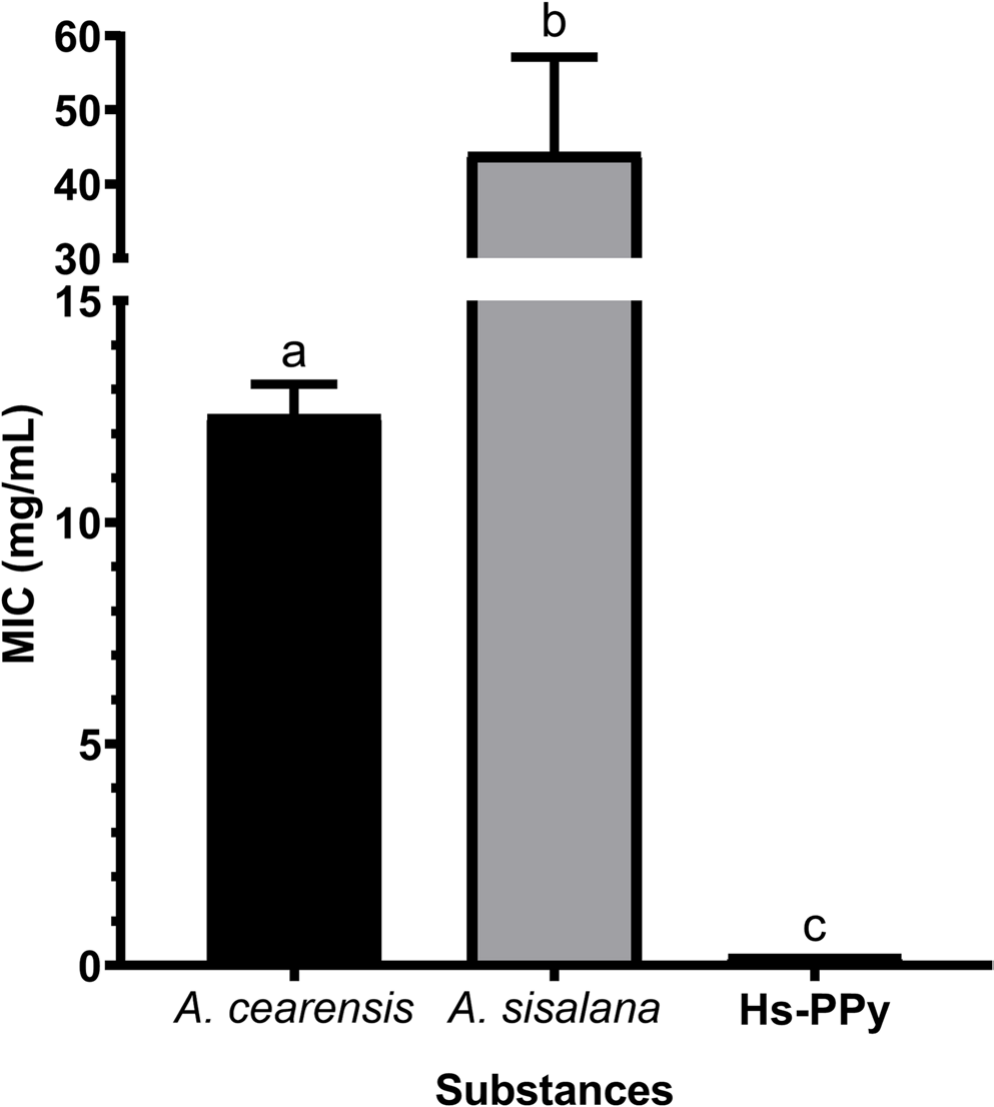
Comparison of minimum inhibitory concentration (MIC) results for *Amburana cearensis* and *Agave sisalana* extracts and highly soluble polypyrrole (Hs-PPy) against *Escherichia coli* isolates from bovine mastitis. Different letters indicate statistical difference (*P* < 0.05) between treatments

Comparative MIC analyses reveal statistically significant differences among the results obtained for *A. cearensis* and *A. sisalana* extract and Hs-PPy, as shown in Fig. 4. Hs-PPy exhibited superior antimicrobial activity. The absence of *C. leptophloeos* from the figure is due to its lack of inhibitory activity against all the isolates evaluated.

## Discussion

Most isolates were found to be susceptible to the tested antimicrobials, with the highest resistance rate identified for cephalexin. However, resistance to more than one antibiotic is cause for concern [44], and these results may be related to the region and the amount of antimicrobial used in the treatment of mastitis, since the frequency of resistance varies.

In terms of specific function, the *acrA* gene encodes an adapter protein that acts in the periplasm [45]. The *acrB* gene functions as a heterotrimer that forms the inner membrane component and is responsible for substrate recognition and energy transduction, thus acting as a drug/proton antiporter in Gram-negative bacteria [46]. *TolC* is an outer membrane efflux protein that regulates efflux. The combination of these genes comprises the AcrAB-TolC efflux pump, which is part of the Resistance-Nodulation-Division (RND) family [47], with the function of transporting chemical compounds into the bacterial cell [48].

The presence of other families has different strategies of action, but they act with the aim of bacterial persistence in the host, so it is necessary to identify the genes present in these families, with the ATP-linked antibiotic efflux pump that confers solute import/export being found in the genomes [49]. The major facilitator superfamily (MFS) acts on the inner part of the membrane bilayer [50]. The general bacterial porin with reduced permeability to beta-lactams acts by affecting the entry of antibiotics into the inner membrane, thus altering the amount of drug that enters the bacteria [51].

Regarding the other identified efflux pumps, only one isolate exhibited neomycin resistance associated with AcrAD-TolC, which is responsible for aminoglycoside resistance [52], while six exhibited a pattern of intermediate resistance. In Enterobacteriaceae, a pump found primarily in *Klebsiella pneumoniae*, called KpnEF, was detected in *E. coli* isolates. This is cause for great concern, as horizontal gene transfer enables greater resistance in isolates. Among the identified pumps, this is one of those that acts on broad-spectrum antimicrobials [53]. The MdtABC-TolC pump acts against quinolones [54], of which enrofloxacin is the only one tested. Only one isolate was considered resistant. Finally, the presence of the MdtNOP efflux pump was observed, which acts against sulfonamides [55]. Among the drugs tested, sulfazotrim stood out for being considered sensitive in almost all isolates and may be used against bovine mastitis.

The genes related to the virulence of the isolates promote the degree of pathogenicity, thus intensifying the harm to the herd. Among the gene sets present in the genomes are those related to adhesion, an important factor for the onset of host colonization [56]. The specific genes found that may be related to the in vitro results include *fimA*, *fimB*, *fimC*, *fimD*, *fimE*, *fim*F, *fimG*, *fimH*, *fimI*, and *fimZ*. The *fim* genes are frequently found in bovine mastitis isolates and colonization of the mammary epithelium, with these effects generally associated with the *fimA* and *fimH* genes [57]. The genes *csgD*, *csgE*, *csgF*, *csgG*, and *csgC* act on host cell adhesion, thus promoting entry for possible biofilm formation [58, 59].

In addition to these sets, the presence of motility for the bacterial cell is extremely important, enabling it to reach intra- and extracellular locations to acquire nutrients and attach to form biofilms. The related genes found are *cheA*, *cheB*, *cheR*, *cheW*, *cheY*, and *cheZ*, related to the chemotaxis system of *E. coli* [60], enabling movement along the gradient. The genes *flgB*, *flgC*, *flgD*, *flgG*, *flgH*, and *flgI* are involved in flagellum synthesis, which mediates bacterial cell locomotion [61]. Similarly, *fliA*, *fliG*, *fliI*, *fliM*, *fliN*, *fliP* and *fliQ* are responsible for motility; among these, *fliA*, plays a critical role in the formation of bacterial communities at higher temperatures [62, 63].

The results of *A. cearensis* at a tested concentration of 12,500 µg/mL differed from previous studies, which found the plant extract to be effective against *E. coli* at an inhibitory concentration of 1000 µg/mL [64, 65], and against Gram-negative bacteria at 512 µg/mL in synergism with antibiotics [66]. Oliveira et al. [67] highlighted the efficiency of the extract at a concentration of 9,000 µg/mL against ATCC *S. aureus*, but observed no antibacterial activity against ATCC *E. coli*. This lack of efficacy may be explained by the fact that these bacteria were classified as multidrug-resistant, suggesting they may share some resistant mechanisms.

In relation to *C. leptophloeos*, the results are similar to those of Figueredo et al. [68], who found clinically irrelevant MIC values for the bactericidal action of this extract against standard strains of *S. aureus* and *Pseudomonas aeruginosa*. In contrast, *E. coli* was inhibited at a MIC of 12,500 µg/mL [17],. These variations in antimicrobial activity correlate with the specific plant part used; for example, leaf extracts have shown inhibition at a concentration of 3,125 µg/mL, whereas bark extracts were effective at 781.2 µg/mL [18]. The extract’s antibacterial activity against the isolates in this study is supported by the complexity of Gram-negative bacteria, as studies have shown the drug to be ineffective against some isolates [69].

The results for *A. sisalana* extract are in line with studies that have used it, with unsatisfactory results when using the extract alone against Gram-positive and Gram-negative bacteria with different MIC volumes [70]. Hammuel et al. [20] found significant results when using the extract against *S. aureus* and *E. coli* from Nigeria, with an activity range of 10,000 to 12,000 µg/mL.

Hs-PPy was the only compound that showed bactericidal activity, with inhibition occurring at concentrations up to 250 µg/mL. This result is consistent with studies in which the inhibitory concentrations that showed activity were 125 µg/mL and 62 µg/mL in *Staphylococcus* spp. of dairy animals, from Brazil [23]. Studies have already demonstrated the efficacy of Hs-PPy against *S. aureus* at a concentration of 125 µg/mL [25]. Therefore, studies have shown that Hs-PPy did not alter its average concentration of action even against sensitive and resistant isolates, thus demonstrating bacterial inhibition and proving to be an option against resistant isolates of *E. coli* from bovine mastitis. Furthermore, previous research demonstrated that the use of Hs-PPy did not induce changes in milk. Consequently, utilizing this material for mastitis treatment would not negatively affect the final product, posing no risk to the consumer [71]. It is known that the standard polypyrrole has negligible cytotoxicity against fibroblasts [72].

The global health scenario has been facing challenges in dealing with multidrug-resistant bacteria such as *E. coli*, with the dairy industry suffering economic losses due to herds suffering from mastitis. Therefore, to eliminate the bacteria, an effective alternative is needed to bring greater safety to society, requiring research into antimicrobial agents, since the incidence of drug-resistant bacteria in herds and the human population has been recurrent [11, 73].

The limited number of isolates available for this study may be considered a limiting factor for a better interpretation of the species’ genomic data and does not allow for a comprehensive evaluation of the tested compounds; therefore, future studies could evaluate a larger number of isolates.

## Conclusions

In conclusion, the *C. leptophloeos* extract did not exhibit antimicrobial activity. However, Hs-PPy showed superior antimicrobial activity compared to the *A. cearensis* and *A. sisalana* extracts against *E. coli* isolates from bovine mastitis that possessed efflux pumps, virulence genes, and resistance genes. These isolates may be cause for concern because they possess mechanisms for multidrug resistance and have a wide range of virulence genes that contribute to their pathogenicity. Consequently, even in the absence of direct expression or utilization of these genes, they may function as a reservoir for other bacterial species. Therefore, Hs-PPy shows promise as an alternative for controlling *E. coli*, even in the presence of genes that hinder the action of other compounds and may be useful in the treatment of bovine mastitis.

## Data Availability

All data supporting the findings of this study are available within the paper and its Supplementary Information.
